# Analysis of SMALP co-extracted phospholipids shows distinct membrane environments for three classes of bacterial membrane protein

**DOI:** 10.1038/s41598-018-37962-0

**Published:** 2019-02-12

**Authors:** Alvin C. K. Teo, Sarah C. Lee, Naomi L. Pollock, Zoe Stroud, Stephen Hall, Alpesh Thakker, Andrew R. Pitt, Timothy R. Dafforn, Corinne M. Spickett, David I. Roper

**Affiliations:** 1 School of Life Sciences, Gibbet Hill Road, University of Warwick, Coventry, CV4 7AL UK; 2 School of Biosciences, University of Birmingham, Edgbaston, Birmingham, B15 2TT UK; 3 School of Life and Health Sciences, Aston University, Aston Triangle, Birmingham, B4 7ET UK

## Abstract

Biological characterisation of membrane proteins lags behind that of soluble proteins. This reflects issues with the traditional use of detergents for extraction, as the surrounding lipids are generally lost, with adverse structural and functional consequences. In contrast, styrene maleic acid (SMA) copolymers offer a detergent-free method for biological membrane solubilisation to produce SMA-lipid particles (SMALPs) containing membrane proteins together with their surrounding lipid environment. We report the development of a reverse-phase LC-MS/MS method for bacterial phospholipids and the first comparison of the profiles of SMALP co-extracted phospholipids from three exemplar bacterial membrane proteins with different topographies: FtsA (associated membrane protein), ZipA (single transmembrane helix), and PgpB (integral membrane protein). The data showed that while SMA treatment per se did not preferentially extract specific phospholipids from the membrane, SMALP-extracted ZipA showed an enrichment in phosphatidylethanolamines and depletion in cardiolipins compared to the bulk membrane lipid. Comparison of the phospholipid profiles of the 3 SMALP-extracted proteins revealed distinct lipid compositions for each protein: ZipA and PgpB were similar, but in FtsA samples longer chain phosphatidylglycerols and phosphatidylethanolamines were more abundant. This method offers novel information on the phospholipid interactions of these membrane proteins.

## Introduction

Membrane proteins have a central place in the function of the cell: they mediate key processes such as the transfer of ions, nutrients and signals into and out of the cell, and in microorganisms contribute to cell membrane biosynthesis and cell division. In view of the urgent need to develop novel antimicrobial agents, much interest has focused on proteins involved in the latter two processes, often using the Gram-negative bacterium *Escherichia coli* as a model system. For example, ZipA and FtsA act as anchors for the cytoskeletal bacterial tubulin homolog FtsZ, are part of the divisomal apparatus, and migrate to the division septum^1^. ZipA is a bitopic membrane protein harbouring a short N-terminal transmembrane helix^2^, whereas FtsA is thought to attach peripherally to the cell membrane by a short C-terminal amphipathic helix motif^3^. Another important membrane protein, PgpB, is an integral membrane lipid phosphatase with a key role in membrane biosynthesis by generation of phosphatidylglycerol (PG), but additionally with the ability to dephosphorylate the glycan lipid carrier undecaprenyl pyrophosphate^4^. The activity of PgpB depends on the acyl chain length of the lipid substrates and has been reported to be partially inhibited by the zwitterionic phospholipid phosphatidylethanolamine (PE), leading to the suggestion that PE acts as a regulator of PgpB activity to balance the amount of zwitterionic and anionic phospholipids in the membrane necessary for the survival and growth of *E*. *coli*^5,6^. Previous studies have shown that areas of high membrane curvature, such as the septum, contain specific lipids that are essential for correct membrane morphology during division^7^, and for FtsA and ZipA as well as PgpB, the local membrane lipid environment is likely to be critical for their correct structure and function. However, until recently it has been difficult to study them in a native condition or determine the associated lipids, because of the extraction and reconstitution methods commonly used for membrane proteins.

Historically, studies of membrane protein structure and function have typically involved extraction using detergents such as *n*-dodecyl-β-D-maltopyranoside (DDM) that strip away membrane lipids to leave the protein in a detergent micelle. However, over the last 10 years there has been a re-evaluation of this method, as it is becoming clear that studies of membrane proteins should take into account their local environment, including the immediately surrounding lipids^8^. A growing number of studies have shown that membrane proteins associate closely with specific lipids in the membrane, and that this association is important for fundamental aspects of their function, including their folding, activity and interactions^9–12^, but data on which lipids a membrane protein is specifically associated with is still very limited; co-purified membrane lipids have only been detected in a small number of studies of detergent-purified membrane protein, as usually the detergent displaces these lipids^13^. A further difficulty in studying membrane proteins relates to their reconstitution from a detergent micelle into lipid bilayer. Apart from the problem that these artificial liposomes may not accurately reproduce the native lipid environment of the protein, such reconstitutions are often complex and yield samples that can be difficult to study. For example, the presence of lipid bilayers can produce scattering artefacts which require substantial instrumental modifications for many optical techniques, particularly those, like circular dichroism (CD), that operate in the ultraviolet^14^. In addition, native mass spectrometry is developing as a promising method for the detailed analysis of closely associated lipids^15,16^, including in lipoprotein nanodisc systems described below^17^, and there is a growing number of laboratories that have the advanced technology and experience to perform this.

An approach that has made a step-change in the study of membrane proteins is the use of Styrene Maleic Acid/Lipid Particles (SMALPs). The SMALP method uses an amphipathic polymer, styrene maleic acid co-polymer (SMA), to extract membrane proteins directly from native membranes^8^. In contrast to detergent, the SMA reagent extracts the protein within the portion of membrane that surrounds it, thereby in theory preserving the native lipid environment^18^. This assertion has been strengthened by the first high resolution cryo-EM structure of a SMALP solubilised protein, bacterial alternative complex III, which showed the electron densities of several phospholipids tightly bound to the protein, although the precise phospholipid class was not confirmed^19^. Extraction of the lipids from SMALP particles therefore generates the perfect sample for studies of the local lipid environment surrounding a protein. Several studies utilizing SMALP-extracted proteins have involved some form of lipid analysis, including phosphate quantification^20–23^, thin layer chromatography^22–27^, gas chromatography^25,28^, and mass spectrometry (MS)^28–31^. Some studies have reported the enrichment of certain types of lipids with the membrane proteins of interest^22,25,28–30^, highlighting the usefulness of the SMALP approach in studying the protein-associated lipidome. Most studies provided limited detail on the exact lipid species, but some recent work using mass spectrometry has enabled a more thorough profiling of the phospholipids present in SMALPs^30,31^, illustrating the power of this approach to address the question of local lipid environment of membrane proteins.

Therefore, the aim of this study was to carry out the first SMALP-based extraction of ZipA, FtsA, and PgpB from *E*. *coli* membranes, and develop an effective reverse phase liquid chromatography tandem mass spectrometry (LC-MS/MS) method to characterise and compare their co-extracted lipids. A key goal was to determine whether the integral membrane protein (PgpB) associates with different membrane phospholipids to the bitopic protein ZipA and peripheral membrane protein FtsA.

## Results

### Development of a target lipidomics method for use on SMALP samples

The composition of wild type *E*. *coli* K-12 membrane lipids has previously been characterised by LC-MS. Oursel *et al*. used reverse-phase LC-MS/MS to separate PE and phosphatidylglycerol (PG), but cardiolipin (CL; sometimes called di-PG) was not reported^32^, whereas in a later study a normal-phase LC-MS method was employed to examine CL alone^33^. To improve on this, we developed a reverse-phase LC-MS/MS method to separate simultaneouly the three main classes of *E*. *coli* membrane phospholipids: PE, PG and CL. The main challenge was to find a column and solvent system capable of separating PG and CL, which are structurally similar, while also obtaining good ionisation of the phospholipids. This was achieved using a gradient of tetrahydrofuran (THF)/methanol (MeOH)/water (H_2_O) containing 10 mM ammonium acetate on a Luna^®^ C8(2) column. The sensitivity of the method was tested using an exponential dilution (in the concentration range of 0.039–2.5 ng on column, i.e. per injection, per individual lipid) of a lipid mixture containing equal amounts of three synthetic phospholipids: PE(16:0/18:1), PG(16:0/18:1) and CL(18:1/18:1/18:1/18:1). The limit of quantification (LoQ) was determined to be between 0.039–0.078 ng for both PE(16:0/18:1) and PG(16:0/18:1), while CL(72:4) showed a LoQ approximately 10-fold higher at 0.31–0.63 ng (Supplementary Table S1). The LC-MS/MS method developed was also appropriate for the separation and detection of phosphatidylcholines (PC) (Supplementary Fig. 1), which are abundant in eukaryotic membranes and appear to be present in some bacterial species^34^, demonstrating the versatility of this reverse-phase LC-MS/MS method.

**Figure 1:**
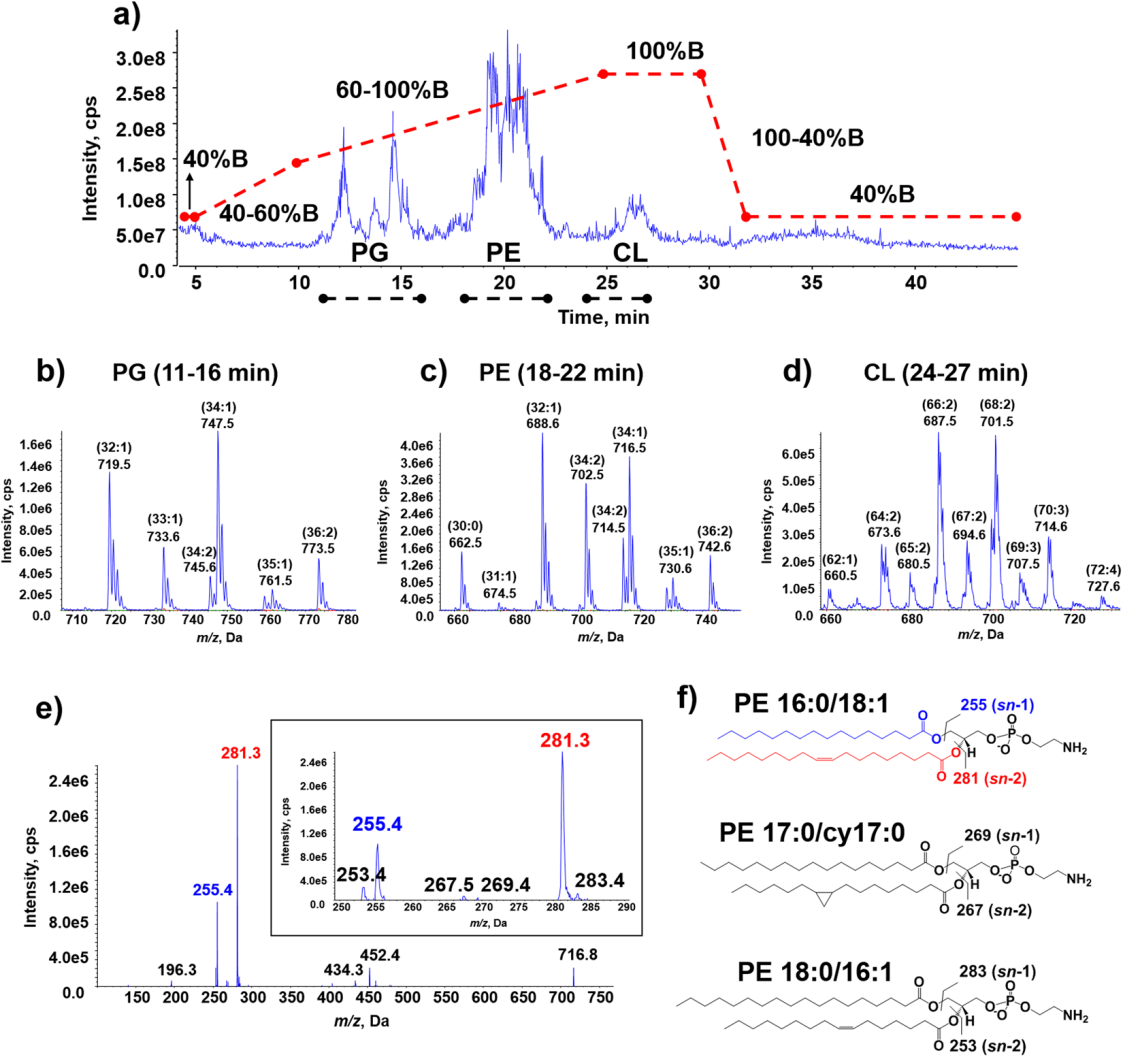
LC-MS/MS analysis of SMALP extracted membrane lipids. (**a**) TIC of SMALP extracted *E*. *coli* BL21(DE3) membrane lipids on the Luna^®^ C8(2) column under a THF/MeOH/H_2_O gradient. Dotted lines (red) indicate the change in gradient as solvent B percentage and typical elution time windows for the three membrane phospholipids of *E*. *coli* (PG, PE, and CL) are indicated with dotted lines (black). (**b**–**d**) Combined negative ion mass spectra across these regions showing the variety of PG (**b**), PE (**c**), and CL (**d**) phospholipids. The phospholipids are labelled as *C*:*n*, whereby *C* = number of carbons in the fatty acyl chains; *n* = double bond equivalents. Theoretical masses for the lipids can be found in Supplementary Table S2. (**e**) Product ion (MS/MS) scan in negative ion mode of PE 34:1 (*m/z* 716) showing the fragmentation of this species to yield different fatty acyl chains (insert, *m/z* 250–290). (**f**) The potential structures of molecular species of PE(34:1), of which 16:0_18:1 was the most abundant species.

The method was then applied to investigate the extraction of *E*. *coli* BL21(DE3) membrane lipids. A representative total ion chromatogram (TIC) illustrating the effective separation of the membrane lipids is shown in Fig. 1a, and it can be seen that the phospholipids eluted based on hydrophobicity with the elution order: PG > PE > CL. Figure 1b–d show the variety of molecular phospholipid species that were detected in each region of the chromatogram. The identity of the phospholipids were initially assigned using their nominal mass-to-charge ratio (*m/z*), with PE occurring at even *m/z* values, and PG and CL occurring at odd *m/z* values; the CL species were doubly charged and could be distinguished from the PG species by the spacing of the isotopic peaks. To confirm the identity and provide structural characterisation of the different molecular species, tandem MS in negative ion mode was employed; an example spectrum is shown in Fig. 1e for one of the most abundant PE species (PE(34:1)) at *m/z* 716. Based on the fatty acyl chain fragmentation products, it was determined to be comprised of three different *sn*-positional isomers: PE(16:0_18:1) (fragment ions at *m/z* 255 and 281), PE(17:0_17:1) or PE(17:0_cy17:0) (fragment ions at *m/z* 269 and 267) and PE(18:0_16:1) (fragment ions at *m/z* 283 and 253). The structures and corresponding fatty acyl (RCOO^−^) ion masses are shown in Fig. 1f and a full list of phospholipid species and their m/z is given in Supplementary Table S2).

### Do lipids extracted using SMALPs match those found in the membrane?

**Figure 2:**
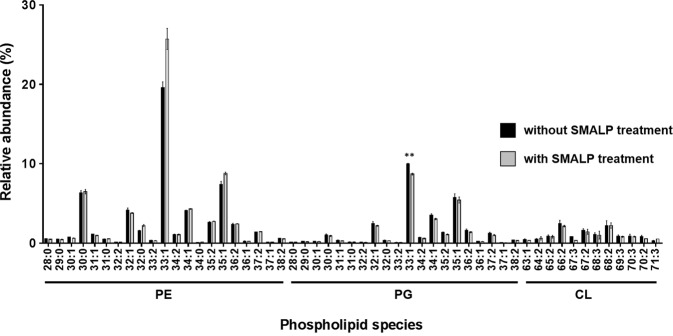
Comparison of the phospholipid profiles of *E*. *coli* BL21(DE3) SMALP vs non-SMALP (multiple *t*-tests with Bonferroni correction). A ssignificant difference is denoted as **(*p* < 0.01) for PG(33:1). Data points correspond to three technical replicates. Error bars represent ± S.D.

An important consideration for the use of SMALPs to sample the lipids in a membrane is the assumption that the SMA-extraction process has no selectivity for any particular lipid. To determine whether this was the case and therefore that SMALPs are a suitable tool for lipidomic studies, *E*. *coli* BL21(DE3) membranes were separated into two fractions, one of which was subjected to SMA-treatment to generate generic membrane SMALPs prior to lipid extraction, and one in which the lipids were extracted directly without SMA-treatment. The lipids extracted from both fractions were analysed using LC-MS/MS. As MS is not inherently quantitative, three non-natural lipid internal standards, PE(12:0/13:0), PG(12:0/13:0) and CL(14:1/14:1/14:1/15:1), were added to the samples prior to the lipid extraction to allow downstream data normalisation and relative quantification of each phospholipid class. Figure 2 demonstrates that the overall phospholipid profiles for extracted BL21(DE3) membrane phospholipids with or without SMA treatment were highly comparable, with no major depletion of specific lipid types in the SMA-treated sample. The only significant difference was a small decrease in PG(33:1) in the SMALP sample. This suggests that, at least for the *E*. *coli* membranes used here, the SMA copolymer does not preferentially extract certain types of membrane lipids, which agrees with previous studies by other groups who have studied the solubilisation of *E*. *coli* membrane lipids by the SMA copolymer^25,35,36^.

### Effect of induction of ZipA expression on the *E. coli* lipidome

**Figure 3:**
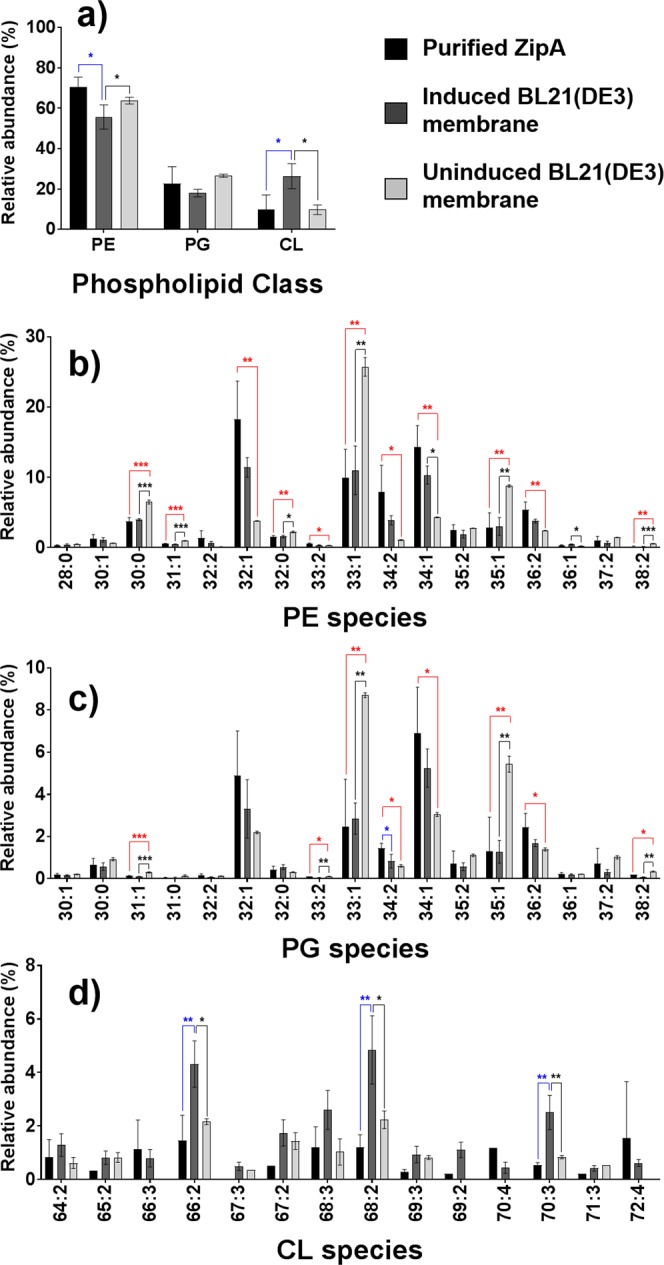
Comparison of the lipid profiles of the SMALP-purified ZipA with its respective IPTG-induced and uninduced membrane controls. The phospholipid compositions are either shown as sum for each of the three classes (**a**), or as individual molecular species under PE (**b**), PG (**c**) or CL (**d**). All phospholipid species were confirmed and assigned by tandem MS in negative ion mode. Data points for purified ZipA and induced BL21(DE3) membrane represent three biological replicates (prepared from different cultures), while for uninduced BL21(DE3) three technical replicates (derived from the same culture to show reproducibility of the analytical method) were carried out. Error bars represent ± S.D. Significant differences (upon one-way ANOVA) are denoted as *(*p* < 0.05), **(*p* < 0.01), or ***(*p* < 0.001).

It has been reported previously that the lipidome can change in response to induction of protein expression^37^. To examine the effects of protein expression on the total lipid present in the bulk membrane of *E*. *coli*, the lipid profiles of BL21(DE3) cells harbouring the ZipA plasmid with or without IPTG induction were determined using LC-MS/MS. Approximately 50 phospholipids of distinct *m/z* could be detected (without taking into account isomeric species) in all 3 samples (Fig. 3). In uninduced cells the relative abundance of lipids containing double bond equivalents (dbe, containing either unsaturation or a ring) varied considerably between classes, with lipids containing a single dbe (32:1, 33:1, 34:1 and 35:1) being most abundant for PE and PG, whereas for CLs, species with multiple dbe, such as 66:2, 68:2 and 70:3, were more abundant. In the bulk membrane, induction of ZipA expression significantly increased the total proportion of CL while decreasing the amount of PE in the membrane (Fig. 3a). However, closer examination of the individual lipids showed that IPTG induction appeared to decrease the amount of the odd numbered, 1 dbe species of PE and PG (31:1, 33:1 and 35:1), a decrease in PE(30:0), and an increase in even numbered one or two dbe PE and PG species (particularly 32:1, 34:2, 34:1 and 36:2). For CL, ZipA induction appeared to increase the amounts of CL(66:2), CL(68:2) and CL(70:3) (Fig. 3d).

The profile of lipids co-extracted with ZipA in SMALPs from the overexpressing cells was also investigated. SMALP-purified ZipA in this system showed an overall class profile most similar to the membrane from uninduced cells, but within the PE and PG classes the profile was most similar to that of the ZipA induced membrane, whereas the CL profile remained most similar to the uninduced membrane. This suggests that individual proteins associate preferentially with a subset of the lipids present in the overall membrane, which can be co-extracted with the protein in SMALPs.

### Comparison of the SMALP lipidome of ZipA, FtsA and PgpB

**Figure 4:**
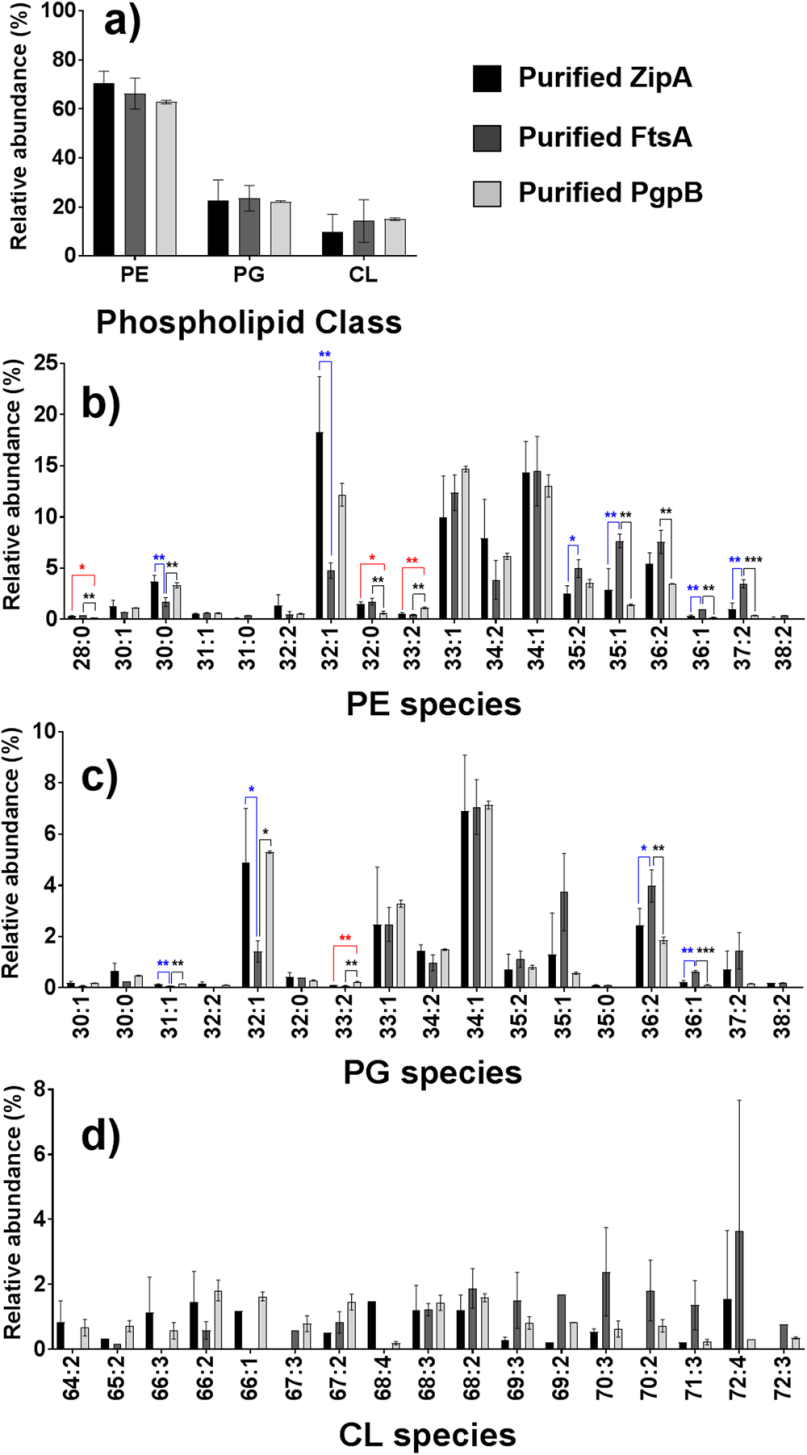
Comparison of the SMALP lipid profiles of the three purified *E*. *coli* membrane proteins: ZipA, FtsA, and PgpB. The phospholipid compositions are either shown as sum for each of the three classes (**a**), or as individual molecular species under PE (**b**), PG (**c**) or CL (**d**). All phospholipid species were confirmed and assigned by tandem MS in negative ion mode. Data points for ZipA and FtsA correspond to three biological replicates, while PgpB corresponds to three technical replicates. Error bars represent ± S.D. Significant differences (upon one-way ANOVA) are denoted as *(*p* < 0.05), **(*p* < 0.01), or ***(*p* < 0.001).

To investigate whether the phospholipids preferentially extracted with other membrane proteins were the same, SMALP co-extracted membrane lipids from FtsA and PgpB were examined using the same LC-MS/MS method and compared to those found with SMALP-ZipA. For all three proteins, good enrichment after IMAC was achieved (Supplementary Fig. 2). At the level of the phospholipid classes, no significant differences between the three *E*. *coli* membrane proteins were observed (Fig. 4a). Nevertheless, significant differences were observed between the phospholipid profiles in the PE and PG classes. The phospholipid profiles of ZipA and PgpB were similar, showing a higher abundance in monounsaturated phospholipid species in both the PE and PG classes. ZipA and FtsA showed substantial differences in their phospholipid profiles: PE(30:0), PE(32:1), and PG(32:1) were more abundant in ZipA, especially PE(32:1) and PG(32:1). In contrast, PE(35:2), PE(35:1), PE(36:1), PE(37:2), PG(36:2), and PG(36:1) were observed to have higher abundance in FtsA.

## Discussion

In this work, we showed that *E*. *coli* membrane proteins ZipA, FtsA and PgpB are amenable to the solubilisation and purification using the 2:1 SMA copolymer (Supplementary Fig. 2); to the best of our knowledge, the work on PgpB represents the first report to-date of an integral membrane lipid phosphatase directly extracted and examined in a detergent-free membrane mimetic system. The solubilisation efficiency of the 2:1 SMA copolymer was previously reported to be comparable to DDM (around 55%) for several other bacterial membrane proteins overexpressed in *E*. *coli*, namely ZipA, BmrA (multidrug efflux pump from *Bacillus subtilis*), and LeuT (amino acid:Na^+^ symporter from *Aquifex aeolicus*)^38^. Previously, lower overall yields of purified protein have been noted when employing the SMALP technology^18^, suggesting a requirement for future improvement in the protein solubilisation and purification protocol. Several other variants of the SMA copolymers which differ in the S:MA ratio and molecular weight were also evaluated by Morrison *et al*.^38^, but the 2:1 variant gave the best yield, purity and functional conservation of target membrane proteins upon a single step of IMAC purification.

Importantly, in this work the SMA copolymer was found to not preferentially solubilise specific types of *E*. *coli* membrane phospholipids. Similar observations have also been reported for the cell membranes of *R*. *sphaeroides*^23^, *S*. *cerevisiae* mitochondria^26,27^ and synthetic PC/PE large unilamellar vesicles^39^ using the SMA copolymer. This fact highlights the utility of the SMA copolymer as an alternative to detergents for biological membrane solubilisation, which facilitates subsequent membrane lipid characterisation without selective lipid bias, at least for *E*. *coli*, which is a common host for recombinant protein production.

A key finding of this work was that significant differences were observed between the phospholipid profiles in the PE and PG classes for the three *E*. *coli* membrane proteins. The phospholipid profiles of ZipA and PgpB were similar, showing a higher abundance in monounsaturated phospholipid species in both the PE and PG classes, possibly because both of these proteins are embedded in the lipid membrane, either through a single transmembrane helix (ZipA) or fully embedded with six transmembrane helices (PgpB), whereas FtsA is a peripheral membrane protein.

FtsA has been reported to interact with membrane lipids mainly through hydrophobic interactions^40^. Recently, Conti *et al*. reported the purification of IPTG-induced, overexpressed FtsA in membrane lipid vesicles using a native (detergent-free) purification technique^41^. The interaction between phospholipids and FtsA was suggested to be essential for rapid ATP hydrolysis *in vitro*. FtsA and other peripheral membrane proteins, such as SpoVM and DivIVA (two curvature-sensitive proteins in *B*. *subtilis*), have been suggested to associate with the cell membrane by inserting the hydrophobic residues of their proposed amphiphatic helix into the cell membrane^41,42^. Possibly the higher abundance of phospholipids with longer fatty acyl chains in FtsA (35–37 carbons vs 32 carbons in ZipA) might be required to facilitate this. Given that the sum of each of the phospholipid classes were similar for these three membrane proteins, yet significant differences were observed in terms of different fatty acyl profiles, this suggests a clear preference for different lipid chain lengths and/or degree of unsaturation (rather than headgroup) for these bacterial membrane proteins, which is an important novel finding.

It is known that the annular lipid belt of proteins can undergo exchange with the bulk lipids of the biological membrane. In the absence of any selectivity between the lipids and protein, the annular belt largely reflects the composition and properties of the surrounding membrane^43^. Recent publications on the exchange of lipids in SMALPs (without membrane proteins) have suggested that conclusions cannot be drawn on particular lipid enrichment of the target proteins unless they are tightly bound to the proteins^39,44,45^. The 2:1 SMA copolymer was found to have a much slower collisional lipid transfer than the 3:1 counterpart, owing to higher coloumbic repulsion^46^. So far, no evidence of lipid transfer has been reported for SMALP-protein nanodiscs. In the future, different SMA variants could be investigated to tune the size of the SMALP nanodiscs that encapsulate varying amount of membrane lipids, to examine the lipids that are most closely associated with the target membrane proteins. Alterntively, complementary studies using native MS could be employed to screen for the most tightly bound lipids upon controlled delipidation using detergents^47^.

An interesting additional finding of our work was that changes in total membrane lipid composition occurred when ZipA was overexpressed in *E*. *coli*. However, the observed increase in CL content did not seem to be locally associated with ZipA, as there was no significant difference between the CL profile for the SMALP-purified ZipA and the uninduced membrane control. The overexpression of ZipA^48^ and FtsA^41^ has been reported to result in multilayered membrane inclusions within the cytoplasm, and similar extra membrane structures have been observed following overexpression of several other membrane proteins, including the F_1_F_o_ ATP synthase, fumarate reductase, glycerophosphate acyltransferase PlsB, mannitol permease MltA, alkane hydrolase AlkB, chemotaxis receptor Tsr, and lipid glycosyltransferases^49^. Thus overexpression of the target membrane proteins using conventional IPTG-induction techniques may represent a limitation of this work, as the co-extracted lipids might not represent the exact spatio-temporal picture of the local lipid environment of the target proteins when *in vivo* in wild-type cells. Nevertheless, specific lipid enrichment has been reported in other SMALP studies^25,30^, indicating that the SMALP co-extracted lipidomes are not merely a snapshot of the native biological membrane at the time of solubilisation, but can indeed reflect specific protein-lipid interactions. This supports the use of SMALP technology as a valid means to probe protein-lipid interactions.

Together, the data reported here show how the combination of MS lipidomic analysis and SMALP encapsulation provides an analytical method that is capable of both qualitative and quantitaive analysis of the protein-associated lipidome for any sample that can be SMA solubilised and enriched using a suitable affinity method (e.g. recombinant tags or protein specific antibodies), could be applied to any membrane protein sample under investigation. Moreover, it demonstrates for the first time that 3 membrane proteins of different types associate preferentially with specific phospholipid profiles.

## Methods and Materials

Unless stated otherwise, all chemicals and reagents used were purchased from Sigma-Aldrich (Dorset, UK), Fisher Scientific (Leicestershire, UK), or Melford Biolaboratories (Suffolk, UK). All organic solvents used in the MS experiments were of HPLC grade and LC-MS grade H_2_O was used.

### Membrane protein overexpression and purification

*E*. *coli* ZipA and FtsA were overexpressed and purified based on methods of Lee *et al*.^18^ and PgpB based on Touzé *et al*.^6^. The ZipA and FtsA proteins were purified from *E*. *coli* BL21(DE3) cells following overnight incubation at 25 °C post IPTG induction, while the PgpB was purified from *E*. *coli* C43(DE3) cells after 4 h incubation at 37 °C post IPTG induction. In outline, the bacterial cells were lysed using steps 1–5 of the protocol of Harder and Fotiadis^50^. Cells were lysed using a French press, unlysed cells removed by centrifugation at 10,000 g, and the membranes then pelleted by centrifugation at 100,000 g. Membranes were washed once in lysis buffer and once with buffered 300 mM NaCl (to remove adherent proteins) by resuspension and homogenization followed by centrifugation at 100,000 g. The pellet was then fully resuspended (using a Potter homogenizer) in the buffer for SMALP extraction. The overexpressed ZipA, FtsA and PgpB were then solubilised by addition of SMA 2000 to a final concentration of 2.5% (prepared as per Lee *et al*.^44^). The clarified SMALP preparations were centrifuged at 100,000 g at 4 °C for 45 min to removed the insoluble fraction and produce a purified SMALP preparation. Samples were taken at this stage for analysis of total SMALP-extracted membrane lipid. The SMALP extracted ZipA and FtsA were subsequently purified by nickel-nitrilotriacetic acid immobilised metal affinity chromatography (Ni-NTA IMAC) (Generon, UK or Qiagen, USA) followed by size exclusion chromatography, while only Ni-NTA IMAC was used to purified the SMALP extracted PgpB. The purified proteins were analysed by 12% sodium dodecyl sulfate polyacrylamide gel electrophoresis (SDS-PAGE) (Supplementary Fig. 2). Protein concentrations were estimated using either the A280 method on a NanoDrop ND-1000 spectrophotometer or the Pierce bicinchoninic acid protein assay (Thermo Scientific, USA).

### Lipid extraction

Three synthetic phospholipids, PE(12:0/13:0), PG(12:0/13:0), and CL(14:1/14:1/14:1/15:1) (Avanti Polar Lipids, USA) were used as the internal standards (IS) for the lipidomic analysis. The IS mixture used was made up of either 30 µg/mL (10 µg of PE 25:0, PG 25:0, CL 57:4 each), or 50 µg/mL (10 µg PE 25:0, 10 µg PG 25:0, 30 µg CL 57:4) in MeOH supplemented with 0.005% w/v butylated hydroxytoluene (BHT). The IS mixture (40–100 µL) was added to the purified protein samples or *E*. *coli* BL21(DE3) membranes prior to lipid extraction. Lipid extraction was carried out based on a modified Folch method^51^. Firstly, 200 µL ice-cold MeOH (with 0.005% w/v BHT) was added to 400 µL sample (containing IS), followed by 400 µL ice-cold chloroform (CHCl_3_) and lastly 150 µL ice-cold ultrapure H_2_O. Each sample was vortexed for 20 s and sonicated in ice bath for 15 min, after each solvent addition to facilitate rigorous mixing and extraction. The samples were centrifuged at 16,000 × g for 5 min at 4 °C to achieve phase separation. The lipid-containing (lower) organic layer was collected and the remaining aqueous phase was subjected to a second extraction by adding 400 µL ice-cold CHCl_3_. The samples were centrifuged again at 16,000 × g for 5 min at 4 °C and the organic layer was collected. The resulting lipid extract was dried under a gentle stream of N_2_ gas and stored at −20 ^o^C until analysis.

### Liquid chromatography-tandem mass spectrometry (LC-MS/MS)

Samples were reconstituted in 1 mL of 9:1 v/v MeOH:CHCl_3_, followed by appropriate dilution (typically 2 or 5-fold) in the chromatography starting solvent detailed below. Ten µL of reconstituted lipid extract was injected onto a Luna C8(2) 100 Å column, 150 × 1 mm (length × internal diameter), 3 µm (particle size) (Phenomenex, USA) at a flow rate of 50 µL/min solvent A. Lipid separation was achieved using a gradient of the following mobile phases: THF:MeOH:H_2_O (Solvent A: 3:2:5 v/v/v; Solvent B: 7:2:1 v/v/v) containing 10 mM ammonium acetate. The gradient was formulated as follows: 40% B for 4 min, 40% B to 60% B over 6 min, 60% B to 100% B over 15 min, and hold 100% B for 5 min, 100% B to 40% B over 2 min, then reequilibrated to 40% B over 13 min. A QTRAP 5500 mass spectrometer (AB Sciex, UK) was utilised with instrument parameters as follows: curtain gas: 35 psi, ion spray voltage: −4.5 kV (negative ion mode); temperature: 150 °C; nebuliser gas (GS1): 13 psi; auxiliary/turbo gas (GS2): 0 psi; declustering potential: 50 V; entrance potential: 10 V; collision energy: 10 eV. Information-dependent acquisition (IDA) was employed to obtain MS/MS spectra during the LC run for structural characterisation of the lipid species in the negative ion mode. A general survey scan in the EMS mode (with dynamic fill time) at *m/z* 400–1000 Da was used to trigger IDA on one to two most intense ion peak(s) based on the following selection criteria: over *m/z* of 400 Da, 1000 cps, and 20 s; maximum occurrence of 2; mass tolerance of 250 mDa; excluding isotopes within 4 Da. This was followed by enhanced product ion (EPI) scans using the linear ion trap with a collision energy of 45 eV in negative ion mode.

### Data analysis

Mass spectra inspection and data extraction involving relevant peak integration with Gaussian smoothing width of 0–2 points on each extracted ion chromatogram (XIC) were performed manually using the PeakView 2.2 software (AB Sciex, UK). The identification and assignment of each phospholipid species and corresponding molecular structures were facilitated by the IDA LC-MS/MS analysis with subsequent data matching to the LIPID MAPS Structure Database http://www.lipidmaps.org/data/databases.html ^52^. A two-step normalisation approach was employed. Firstly, the peak intensity for each phospholipid species was normalised against the peak intensity for the corresponding class-specific internal standard. This data was then further normalised against the total phospholipid ion intensity per sample/replicate or amount of protein for which lipid extraction was performed to facilitate cross-sample comparison.

### Statistical analysis

Statistical analysis as implemented in either in Microsoft Excel or Prism 7 (GraphPad Software, USA), including unpaired *t*-tests: single or multiple (with Bonferroni correction) and one-way ANOVA with Tukey’s multiple comparisons test.

## Supplementary information

Supplemetry file

## Electronic supplementary material

**Supplementary information** accompanies this paper at 10.1038/s41598-018-37962-0. The Open Access dataset is available at 10.17036/researchdata.aston.ac.uk.00000393.
