# Supplementary material for: Analysis of SMALP co-extracted phospholipids shows distinct membrane environments for three classes of bacterial membrane protein
Source: Sci Rep. 2019 Feb 12;9:1813. doi: 10.1038/s41598-018-37962-0 (PMC6372662; doi:10.1038/s41598-018-37962-0)
Supplement: Supplementary file 1 — Supplemetry file [file 41598_2018_37962_MOESM1_ESM.docx]

**Supplementary Information for:**

**Analysis of SMALP co-extracted phospholipids shows distinct membrane environments for three classes of bacterial membrane protein**

Alvin C. K. Teo^1^, Sarah C. Lee^2^, Naomi L. Pollock^2^, Zoe Stroud^2^, Stephen Hall^2^,Alpesh Thakker^3^, Andrew R. Pitt^3^, Timothy R. Dafforn^2^, Corinne M. Spickett^3^*, David I. Roper^1^*

^1^School of Life Sciences, Gibbet Hill Road, University of Warwick, Coventry, CV4 7AL, UK,

^2^School of Biosciences, University of Birmingham, Edgbaston, Birmingham, B15 2TT, UK,

^3^School of Life and Health Sciences, Aston University, Aston Triangle, Birmingham, B4 7ET, UK.

* corresponding authors’ e-mails:

[david.roper@warwick.ac.uk](mailto:david.roper@warwick.ac.uk);

c.m.spickett@aston.ac.uk

**Supplementary Table S1.** Determination of Limit of Quantification (LOQ) by analysis of the Signal-to-noise ratios (S/N) for decreasing amounts loaded onto the column of each of the 3 standard phospholipids: phospatidylethanolamine PE(34:1) (m/z 716.52); phospatidylglycerol PG(43:1) (m/z 747.51) and cardiolipin CL(72:4) (m/z 727.51).

| **PE(34:1)** |  |  |  |
| --- | --- | --- | --- |
| **Amount (ng)** | **Peak** | **Conc (ng)** | **S/N (1 σ)** |
| 0.0390625 | 7.246E+06 | 0.0390625 | 19.000 |
| 0.078125 | 6.891E+06 | 0.078125 | 16.800 |
| 0.15625 | 1.083E+07 | 0.15625 | 22.267 |
| 0.3125 | 1.745E+07 | 0.3125 | 49.933 |
| 0.625 | 3.515E+07 | 0.625 | 95.533 |
| 1.25 | 6.439E+07 | 1.25 | 195.433 |
| 2.5 | 1.256E+08 | 2.5 | 447.067 |
|  |  |  |  |
| **PG(34:1)** |  |  |  |
| **Amount (ng)** | **Peak** | **Conc (ng)** | **S/N (1 σ)** |
| 0.0390625 | 3.786E+06 | 0.0390625 | 16.400 |
| 0.078125 | 6.160E+06 | 0.078125 | 18.300 |
| 0.15625 | 1.193E+07 | 0.15625 | 28.633 |
| 0.3125 | 2.221E+07 | 0.3125 | 62.100 |
| 0.625 | 4.345E+07 | 0.625 | 103.033 |
| 1.25 | 8.421E+07 | 1.25 | 315.800 |
| 2.5 | 1.581E+08 | 2.5 | 359.067 |
|  |  |  |  |
| **CL(72:4)** |  |  |  |
| **Amount (ng)** | **Peak** | **Conc (ng)** | **S/N (1 σ)** |
| 0.0390625 | NA | 0.0390625 | NA |
| 0.078125 | NA | 0.078125 | NA |
| 0.15625 | NA | 0.15625 | NA |
| 0.3125 | NA | 0.3125 | NA |
| 0.625 | 7.062E+06 | 0.625 | 21.250 |
| 1.25 | 1.469E+07 | 1.25 | 39.667 |
| 2.5 | 2.740E+07 | 2.5 | 87.900 |

**Supplementary Table S2.**  List of phospholipid identifications (total number of carbons in fatty acyl chains: total number of double bonds in fatty acyl chains) based on mass-to-charge ratio (m/z). The theoretical m/z of each species is also given. *IS and italics indicates an internal standard*.

| **Class** | **Molecular species** | | **m/z** | **m/z** |
| --- | --- | --- | --- | --- |
|  |  |  | **([M-H^+^]^-^ measured)** | **([M-H^+^]^-^ theoretical)** |
| *Phosphatidylethanolamine (IS)* | *PE 592* | *PE(25:0)* | *592.4* | *592.3984* |
| Phosphatidylethanolamine | PE 606 | PE(26:0) | 606.4 | 606.4140 |
| Phosphatidylethanolamine | PE 620 | PE(27:0) | 620.4 | 620.42965 |
| Phosphatidylethanolamine | PE 632 | PE(28:1) | 632.4 | 632.42965 |
| Phosphatidylethanolamine | PE 634 | PE(28:0) | 634.5 | 634.4453 |
| Phosphatidylethanolamine | PE 646 | PE(29:1) | 646.5 | 646.4453 |
| Phosphatidylethanolamine | PE 648 | PE(29:0) | 648.5 | 648.46095 |
| Phosphatidylethanolamine | PE 658 | PE(30:2) | 658.5 | 658.4453 |
| Phosphatidylethanolamine | PE 660 | PE(30:1) | 660.5 | 660.46095 |
| Phosphatidylethanolamine | PE 662 | PE(30:0) | 662.5 | 662.4766 |
| Phosphatidylethanolamine | PE 672 | PE(31:2) | 672.5 | 672.46095 |
| Phosphatidylethanolamine | PE 674 | PE(31:1) | 674.5 | 674.4766 |
| Phosphatidylethanolamine | PE 676 | PE(31:0) | 676.5 | 676.49225 |
| Phosphatidylethanolamine | PE 686 | PE(32:2) | 686.5 | 686.4766 |
| Phosphatidylethanolamine | PE 688 | PE(32:1) | 688.5 | 688.49225 |
| Phosphatidylethanolamine | PE 690 | PE(32:0) | 690.5 | 690.5079 |
| Phosphatidylethanolamine | PE 700 | PE(33:2) | 700.5 | 700.49225 |
| Phosphatidylethanolamine | PE 702 | PE(33:1) | 702.5 | 702.5079 |
| Phosphatidylethanolamine | PE 704 | PE(33:0) | 704.5 | 704.52355 |
| Phosphatidylethanolamine | PE 714 | PE(34:2) | 714.5 | 714.5079 |
| Phosphatidylethanolamine | PE 716 | PE(34:1) | 716.5 | 716.52355 |
| Phosphatidylethanolamine | PE 718 | PE(34:0) | 718.5 | 718.5392 |
| Phosphatidylethanolamine | PE 728 | PE(35:2) | 728.5 | 728.52355 |
| Phosphatidylethanolamine | PE 730 | PE(35:1) | 730.5 | 730.5392 |
| Phosphatidylethanolamine | PE 732 | PE(35:0) | 732.6 | 732.55485 |
| Phosphatidylethanolamine | PE 742 | PE(36:2) | 742.5 | 742.5392 |
| Phosphatidylethanolamine | PE 744 | PE(36:1) | 744.6 | 744.55485 |
| Phosphatidylethanolamine | PE 756 | PE(37:2) | 756.6 | 756.55485 |
| Phosphatidylethanolamine | PE 758 | PE(37:1) | 758.6 | 758.5705 |
| Phosphatidylethanolamine | PE 770 | PE(38:2) | 770.6 | 770.5705 |
|  |  |  |  |  |
| *Phosphatidylglycerol (IS)* | *PG 623* | *PG(25:0)* | *623.4* | *623.3930* |
| Phosphatidylglycerol | PG 651 | PG(27:0) | 651.4 | 651.4243 |
| Phosphatidylglycerol | PG 663 | PG(28:1) | 663.4 | 663.4243 |
| Phosphatidylglycerol | PG 665 | PG(28:0) | 665.4 | 665.43995 |
| Phosphatidylglycerol | PG 677 | PG(29:1) | 677.4 | 677.43995 |
| Phosphatidylglycerol | PG 679 | PG(29:0) | 679.5 | 679.4556 |
| Phosphatidylglycerol | PG 691 | PG(30:1) | 691.5 | 691.4556 |
| Phosphatidylglycerol | PG 693 | PG(30:0) | 693.5 | 693.47125 |
| Phosphatidylglycerol | PG 705 | PG(31:1) | 705.5 | 705.47125 |
| Phosphatidylglycerol | PG 707 | PG(31:0) | 707.5 | 707.4869 |
| Phosphatidylglycerol | PG 717 | PG(32:2) | 717.5 | 717.47125 |
| Phosphatidylglycerol | PG 719 | PG(32:1) | 719.5 | 719.4869 |
| Phosphatidylglycerol | PG 721 | PG(32:0) | 721.5 | 721.50255 |
| Phosphatidylglycerol | PG 731 | PG(33:2) | 731.5 | 731.4869 |
| Phosphatidylglycerol | PG 733 | PG(33:1) | 733.5 | 733.50255 |
| Phosphatidylglycerol | PG 745 | PG(34:2) | 745.5 | 745.50255 |
| Phosphatidylglycerol | PG 747 | PG(34:1) | 747.5 | 747.5182 |
| Phosphatidylglycerol | PG 759 | PG(35:2) | 759.5 | 759.5182 |
| Phosphatidylglycerol | PG 761 | PG(35:1) | 761.5 | 761.53385 |
| Phosphatidylglycerol | PG 763 | PG(35:0) | 763.6 | 763.5495 |
| Phosphatidylglycerol | PG 773 | PG(36:2) | 773.5 | 773.53385 |
| Phosphatidylglycerol | PG 775 | PG(36:1) | 775.6 | 775.5495 |
| Phosphatidylglycerol | PG 787 | PG(37:2) | 787.6 | 787.5495 |
| Phosphatidylglycerol | PG 789 | PG(37:1) | 789.6 | 789.56515 |
| Phosphatidylglycerol | PG 801 | PG(38:2) | 801.6 | 801.56515 |
|  |  |  | **([M-2H^+^]^2-^ measured)** | **([M-2H^+^]^2-^ theoretical)** |
| *Cardiolipin (IS)* | *CL 622* | *CL(57:4)* | *622.4* | *622.39045* |
| Cardiolipin | CL 659 | CL(62:2) | 659.5 | 659.445225 |
| Cardiolipin | CL 660 | CL(62:1) | 660.5 | 660.45305 |
| Cardiolipin | CL 666 | CL(63:2) | 666.5 | 666.45305 |
| Cardiolipin | CL 667 | CL(63:1) | 667.5 | 667.460875 |
| Cardiolipin | CL 672 | CL(64:3) | 672.5 | 672.45305 |
| Cardiolipin | CL 673 | CL(64:2) | 673.5 | 673.460875 |
| Cardiolipin | CL 674 | CL(64:1)) | 674.5 | 674.4687 |
| Cardiolipin | CL 679 | CL(65:3) | 679.5 | 680.4687 |
| Cardiolipin | CL 680 | CL(65:2) | 680.5 | 681.476525 |
| Cardiolipin | CL 681 | CL(65:1) | 681.5 | 681.476525 |
| Cardiolipin | CL 682 | CL(65:0) | 682.5 | 682.48435 |
| Cardiolipin | CL 686 | CL(66:3) | 686.5 | 686.4687 |
| Cardiolipin | CL 687 | CL(66:2) | 687.5 | 687.476525 |
| Cardiolipin | CL 688 | C(66:1) | 688.5 | 688.48435 |
| Cardiolipin | CL 693 | CL(67:3) | 693.5 | 693.476525 |
| Cardiolipin | CL 694 | CL(67:2) | 694.5 | 694.48435 |
| Cardiolipin | CL 699 | CL(68:4) | 699.5 | 699.476525 |
| Cardiolipin | CL 700 | CL(68:3) | 700.5 | 700.48435 |
| Cardiolipin | CL 701 | CL(68:2) | 701.5 | 701.492175 |
| Cardiolipin | CL 706 | CL(69:4) | 706.5 | 706.486675 |
| Cardiolipin | CL 707 | CL(69:3) | 707.5 | 707.4945 |
| Cardiolipin | CL 708 | CL(69:2) | 708.5 | 708.502325 |
| Cardiolipin | CL 713 | CL(70:4) | 713.5 | 713.4945 |
| Cardiolipin | CL 714 | CL(70:3) | 714.5 | 714.502325 |
| Cardiolipin | CL 715 | CL(70:2) | 715.5 | 715.51015 |
| Cardiolipin | CL 716 | CL(70:1) | 716.5 | 716.517975 |
| Cardiolipin | CL 720 | CL(71:4) | 720.5 | 720.502325 |
| Cardiolipin | CL 721 | CL(71:3) | 721.5 | 721.51015 |
| Cardiolipin | CL 726 | CL(72:5) | 726.5 | 726.502325 |
| Cardiolipin | CL 727 | CL(72:4) | 727.5 | 727.51015 |
| Cardiolipin | CL 728 | CL(72:3) | 728.5 | 728.517975 |
| Cardiolipin | CL 729 | CL(72:2) | 729.5 | 729.5258 |
| Cardiolipin | CL 730 | CL(72:1) | 730.5 | 730.533625 |


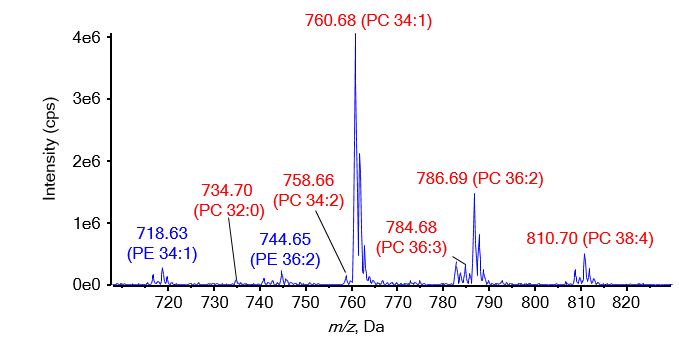


**Supplementary Figure 1.** Mass spectrum of mixed PC (red) and PE (blue) lipids (from soybean) detected using the LC-MS/MS method developed. A variety of PC and PE lipids eluted in the same time window with the method developed. However, isobaric species (molecules with very similar masses that are not fully resolved by the mass spectrometer based on their m/z alone) were still separated using this method. In addition, the application of tandem MS can provide an additional dimension to distinguish the isobaric species based on the characteristic MS/MS fragmentation pattern for these two zwitterionic phospholipids (especially in positive ion mode).


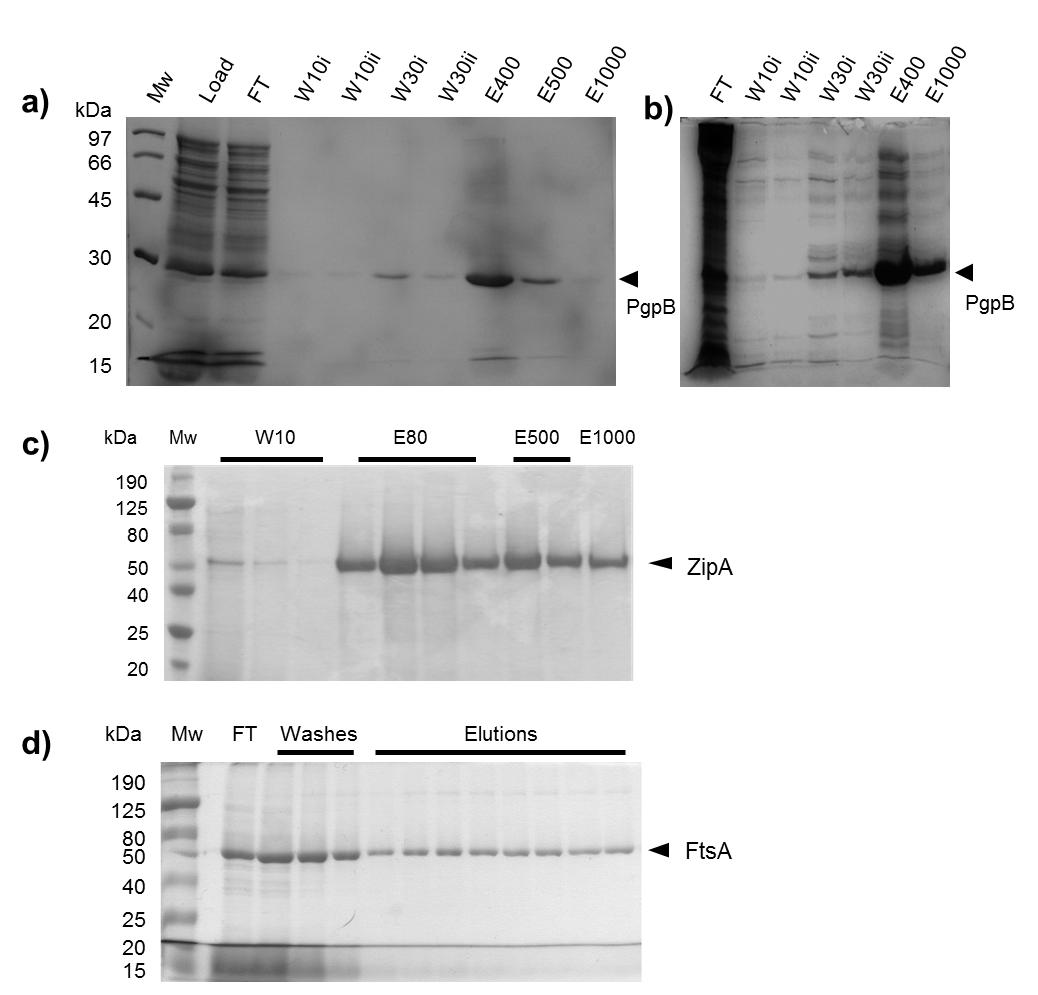


**Supplementary Figure 2.** SDS-PAGE gels showing the purification of proteins extracted using the SMALP approach. (a) SMALP extraction of overexpressed PgpB; b) DDM extraction of PgpB for comparison. Note that upon a single Ni-NTA IMAC purification, PgpB in SMALP was found to be at higher purity than the DDM counterpart. c) SMALP extraction of ZipA showing high purity following a single IMAC purification. Note that Figure 4 in Lee *et al*. ^44^ also illustrated the purity of SMALP-ZipA obtained upon Ni-NTA IMAC and SEC purification; d) SMALP purification of *E. coli* FtsA showing high purity after a single Ni-NTA purification. Legend for all gels: Mw: Molecular weight marker; Load: sample loaded onto the IMAC column; FT: IMAC column flow through; W10i: 10 mM imidazole first wash; W10ii: 10 mM imidazole second wash; W30i: 30 mM imidazole first wash; 30ii: 30 mM imidazole second wash; E400: 400 mM imidazole elution; E500: 500 mM imidazole elution; E1000: 1 M imidazole elution.
